# Validation of the Monocyte Activation Test Demonstrating Equivalence to the Rabbit Pyrogen Test

**DOI:** 10.3390/ijms262211136

**Published:** 2025-11-18

**Authors:** Luisa Burgmaier, Jonas van den Berg, Maria Gajewi, Ruth Röder, Johannes Reich, Sven M. Deutschmann

**Affiliations:** 1 Microcoat Biotechnologie GmbH, Am Neuland 3, 82347 Bernried am Starnberger See, Germany; 2 Department of Thoracic and Cardiovascular Surgery, University Hospital Tuebingen, Calwerstr. 7/1, 72076 Tuebingen, Germany; 3 Roche Diagnostics GmbH, Sandhofer Str. 116, 68305 Mannheim, Germany; 4 Roche Diagnostics GmbH, Nonnenwald 2, 82377 Penzberg, Germany

**Keywords:** Monocyte Activation Test, MAT, pyrogens, endotoxin, non-endotoxin pyrogens, validation

## Abstract

The Monocyte Activation Test (MAT) is an in vitro assay that uses human blood cells to detect both endotoxins and non-endotoxin pyrogens (NEPs), representing a scientifically and ethically superior alternative to the in vivo Rabbit Pyrogen Test (RPT). In the European Pharmacopoeia (Ph. Eur.), the MAT is a compendial method which is explicitly recommended to replace the RPT, requiring only product-specific verification. In contrast, the United States Pharmacopeia (USP) lacks a dedicated MAT chapter, meaning the MAT can only be accepted as an alternative method, given that a full method validation according to USP <1225> is provided in addition to a product-specific verification. This study presents a two-tiered validation strategy addressing both regulatory frameworks: a generic (product-independent) validation aligned with the semi-quantitative test method according to Ph. Eur., ICH Q2, and USP <1225> followed by a product-specific verification. The generic validation consisted of the following parameters: Range and Linearity, Limit of Detection, Accuracy, Specificity, Precision, and Robustness. Robustness was extensively tested under routine-relevant conditions, including variation in the stimulation time, IL-6 read-out timing, freeze–thaw stability, and lot-to-lot comparability. Additionally, an equivalency study of the RPT and the MAT was performed. The data showed that the MAT is at least non-inferior if not superior to the RPT and met the requirements for successful FDA approval of the MAT as an alternative method for pyrogen testing. The MAT was successfully applied to several parenteral drug products. This work provides a transferable framework for GMP-compliant MAT implementation and supports broader international acceptance of the method as a replacement for the RPT in pharmaceutical quality control.

## 1. Introduction

The contamination of parenteral products poses a risk to patient safety. Pyrogens, such as endotoxins from Gram-negative bacteria or non-endotoxin pyrogens (NEPs) from other microbial sources, can trigger systemic inflammation in patients. Endotoxins are lipopolysaccharides of the outer membrane of Gram-negative bacteria, while NEPs include cell wall components from Gram-positive bacteria as well as pyrogens derived from viruses, yeasts, and molds. When a pyrogen-contaminated drug is administered to patients, it can induce fever and, in severe cases, lead to sepsis [1,2,3]. The control and detection of pyrogens is therefore essential and is required by regulatory authorities. As part of the microbial control strategy in pharmaceutical companies, it is common practice to assess the risk of having NEPs present in the manufacturing process. Depending on the risk, a suitable testing strategy must be defined. Historically, parenteral drugs are tested with the in vivo Rabbit Pyrogen Test (RPT) or the Bacterial Endotoxin Test (BET), where the latter is limited to endotoxins [4]. The RPT test involves an intravenous injection of the tested substance into at least three rabbits, which is followed by continuous monitoring of their body temperature over a period of three hours. If the temperature of a single rabbit increases by 0.6 °C or more, additional animals must be tested to confirm the results. The test is qualitative rather than quantitative, meaning it only provides a pass/fail outcome without determining the exact pyrogen concentration [5]. Additionally, the method is highly variable due to biological differences between rabbits and requires up to 12 rabbits per test, making it both costly and ethically controversial [6]. The BET, or more specifically the Limulus Amebocyte Lysate (LAL) test, is a simple and highly sensitive in vitro method for detecting endotoxins. However, it cannot detect non-endotoxin pyrogens, limiting its applicability. Consequently, it has not been able to fully replace the RPT [7,8]. Additionally, the LAL test relies on blood extracted from horseshoe crabs, leading to the annual collection and sacrifice of an estimated 50,000 crabs in the U.S. alone [9]. While a synthetic alternative, recombinant factor C (rFC), mimics the biologicals mechanism of the LAL test without relying on animal blood, this test is not widely used yet [10,11]. Furthermore, the LAL test is subject to interference from high protein levels or beta-glucans in samples.

In 2010, the European Pharmacopoeia (Ph. Eur.) introduced the Monocyte Activation Test (MAT) as a novel in vitro alternative to the RPT, which is capable of detecting both endotoxins and non-endotoxin pyrogens [12]. This introduction marked a significant milestone in the field of pyrogen detection, aligning with the 3Rs principle (Replace, Reduce, Refine) and adhering to the EU Directive 2010/63/EU on animal welfare. The assay is based on the activation of human monocytes, which act as innate immune sentinels through their expression of pattern recognition receptors including Toll-like receptors. Bacterial lipopolysaccharides, for example, are detected via the TLR4/MD-2/CD14 receptor complex, triggering MyD88 and TRIF-mediated intracellular signaling pathways that lead to NF-κB and IRF activation. This signaling induces the transcription and secretion of pro-inflammatory cytokines such as IL-6, IL-1β, and TNF-α. Other microbial pyrogens including lipoproteins, peptidoglycans, viral components, and fungal cell wall polysaccharides are sensed through distinct PRRs such as TLR2, TLR7/8, and dectin-1, enabling broad-spectrum pyrogen recognition. Because the assay measures the direct cellular response of human monocytes, it provides a more physiologically relevant model of pyrogen detection than tests based on non-human systems [13]. Traditionally, MAT has been performed using pooled peripheral blood mononuclear cells (PBMCs) or fresh whole blood, while more recent approaches increasingly rely on standardized cryopreserved preparations or cell lines derived from human monocytes to improve reproducibility and availability. Given the higher sensitivity and physiological relevance of the MAT, its potential for replacing the RPT in routine testing has been widely recognized.

Recognizing the advantages of MAT, the Ph. Eur. now explicitly recommends in vitro tests over in vivo tests whenever patient safety is not compromised. Ph. Eur. Supplement 11.5 consolidated the former MAT formats (Methods A and B) into a single harmonized “Method 1” and redesignated the reference lot comparison as “Method 2” [12]. In parallel, the new general chapter 5.1.13 “Pyrogenicity” [14] provides risk-based guidance on selecting an appropriate assay and confirms that MAT must replace RPT. According to Ph. Eur. chapter 2.6.30 [12], only a product-specific verification is required; a full analytical validation is no longer necessary. This development simultaneously enhances patient safety and significantly reduces animal use in pharmaceutical quality control.

The situation differs in the United States of America, where no USP chapter equivalent to Ph. Eur. 2.6.30 currently exists. Although FDA guidance recognizes “alternative cell-based tests such as the monocyte activation test”, each proposal is reviewed case by case. Pharmaceutical companies must therefore complete a full method validation under USP <1225> [15] in addition to the product-specific verification, which is a requirement that slows widespread MAT adoption for products bound to the U.S. market. Nonetheless, growing international experience and examples such as the work reported here may help pave the way for broader FDA acceptance.

Against this background, this study presents a two tier strategy including a product feasibility study for validating and implementing the MAT in markets where the MAT is not considered a compendial method:

1. Product-independent validation (primary or generic validation)
  At the time of study execution, the former Ph. Eur. 2.6.30 Method B (semi-quantitative format) was still in force, and our generic validation was therefore designed around that method. All parameters specified by Ph. Eur. [16], USP <1225> [15] and ICH Q2 [17]—Range and Linearity, Detection Limit, Accuracy, Specificity, Precision, and Robustness—were examined, with the exception of the quantitation limit, which is not relevant for a semi-quantitative assay. Robustness testing deliberately exceeded formal compendial requirements to demonstrate assay consistency under routine conditions. Additionally, a comparison of the MAT and the RPT was performed using a panel of NEPs. The comparison was based on RPT data from peer-reviewed literature.
  The study assessed the following:
  ○ Range and Linearity of IL-6 read-outs across the working concentration;
  ○ Limit of Detection for endotoxin and representative NEPs;
  ○ Accuracy against spiked reference standards;
  ○ Specificity in the presence of potential interferents;
  ○ Precision (repeatability, intermediate precision, and reproducibility);
  ○ Robustness, which was evaluated by deliberately varying system parameters to assess the assay’s reliability under routine conditions. This included assay components that may differ depending on the specific MAT system used. The following parameters were investigated:
    ▪ Lot-to-lot comparability of the MAT kit;
    ▪ Measurement of the read-out (e.g., IL-6) over time;
    ▪ Stimulation time of the cell source;
    ▪ Freezing and thawing of the cell culture plate after stimulation.
  ○ MAT/RPT comparison
2. Product-specific verification
  The verification is designed to confirm that the drug product does not interfere with pyrogen detection. The verification was performed according to Ph. Eur. 2.6.30 [16].

Hence, the European requirement for “validation for the intended use” and the U.S. requirement for product-specific verification were fulfilled. By detailing this full validation workflow, this paper offers a practical blueprint for laboratories seeking to replace the RPT with MAT while remaining compliant with both European and U.S. expectations. This generic validation, including a direct comparison of the MAT and the RPT, provided the critical data package required for FDA acceptance of the MAT as an alternative method. This paper contributes to the growing body of literature supporting MAT as a scientifically superior, ethically preferable and increasingly harmonized alternative for pyrogen testing.

This study provides a clear roadmap for companies transitioning from the RPT to the MAT using Method 1. It also shows how data collected using the older, now-superseded Method B can be seamlessly applied to the current Method 1 framework. The findings support broader international convergence on MAT, reduce reliance on animal-based tests, and ultimately enhance the safety and efficacy of parenteral medicines.

## 2. Results

### 2.1. Product-Specific Feasibility Study

The product feasibility study is a crucial step in ensuring the reliable detection of endotoxins and NEPs in complex biological or pharmaceutical matrices. It should always be carried out prior to any validation and verification activities. It involves evaluating the feasibility of selected assay conditions, optimizing dilution factors, and identifying potential interferences that may affect assay performance. In this section, we present a case study illustrating how method development may be conducted for one example product.

To establish a robust detection method, the maximum valid dilution (MVD) was calculated according to the equation provided in the Section 4.2. Unless otherwise specified, the endotoxin limit concentration (ELC) for a product is considered equivalent to the contaminant limit concentration (CLC) for the MAT.

A key aspect of method feasibility was the assessment of potential assay interferences. This was evaluated by spiking the product with Reference Standard Endotoxin (RSE) and relevant NEPs, specifically lipoteichoic acid (LTA) and flagellin. Spiked and unspiked samples were analyzed using the MAT according to Ph. Eur. 2.6.30 at dilutions at and below the MVD (i.e., 0.25× MVD, 0.5× MVD, and 1× MVD).

According to Method A, the recovery of spiked pyrogens must fall within 50–200% of the spike recovery in diluent (culture medium). For Method B, the optical density (OD) of the spiked sample must fall between the OD values of the 0.5× and 2× spike concentrations. Table 1 and Table 2 show the measured endotoxin equivalents per mL (EE/mL) values in diluent used for 100% recovery calculation as well as the back-calculated positive product control (PPC) recoveries for the example product (applies also to current Method 1). For all tested dilutions (0.25×, 0.5×, and 1× MVD), spike recoveries for RSE, LTA, and flagellin were within the valid range of 50–200% using Method A. Table 3 and Table 4 present the evaluation according to Method B with all OD values also falling within the acceptance criteria between the 0.5× and 2× spike OD values.

Valid measurements were obtained for all tested dilutions. This applied to RSE as well as to LTA and flagellin spikes, confirming that the sample could be reliably tested using either method with valid recoveries for both endotoxin and non-endotoxin pyrogens.

### 2.2. Generic Method Validation

For the generic method validation, the HaemoMAT^®^ test kit was qualified using an assay setup with a sample-to-spike-to-cell ratio of 1:1:2 (50 µL + 50 µL +100 µL) and a sensitivity of 0.05 EU/mL. The pyrogens included in the validation were RSE, flagellin, LTA, peptidoglycan (PGN), and Pam3CSK4. Please note that the validation results presented herein were conducted according to the former Method B. However, the overall approach remains consistent with the new Method 1.

#### 2.2.1. Range and Linearity

Linearity is defined as the ability of the method to obtain test results that are proportional (either directly or by a well-defined mathematical transformation) to the concentration of the analyte within a given range. To assess linearity, a standard curve was prepared using four endotoxin concentrations ranging from 0.05 EU/mL to 0.4 EU/mL. The linearity of the MAT was evaluated based on the data presented in Section 2.2.4 (Inter-assay Precision). A curve was fitted using a Newton-Raphson 4 parameter logistic function with RSE dilutions. The specific acceptance criteria according to Ph. Eur. chapter 2.6.30 required statistical significance of the regression of responses on a log10 dose with a *p*-value below 0.01, while the regression itself had to show no significant deviation from linearity with a *p*-value above 0.05.

ANOVA analysis was performed using the OD values listed in Table 5. The *p*-value for regression was calculated to 4.4 E-11, which is below the threshold of 0.01, confirming statistical significance. For the deviation of the linearity, the ANOVA determines a *p*-value of 0.99, which was above the required threshold of 0.05. Consequently, the validation parameters for Range and Linearity were successfully met.

#### 2.2.2. Detection Limit

The Detection Limit or Limit of Detection (LOD) refers to the lowest endotoxin concentration that can be reliably detected. According to the former Ph. Eur. chapter 2.6.30 (10th ed. [16]), it is defined as the endotoxin concentration corresponding to the cut-off value. To verify the LOD, the cut-off value must be established by analyzing blank samples in at least two independent assays using two different lots of HaemoMAT^®^ kits. The cut-off value is calculated based on the mean and standard deviation of the blank measurements.

For the method to be considered valid, the signal of the LOQ (OD(RSE 0.050 EU/mL)) has to be above the cut-off value of the blank (corresponds to LOD). The results confirmed that this acceptance criterion was met in all cases. Specifically, the cut-off values (in OD) were determined to be 0.080 and 0.064 for Lot A and 0.037 and 0.052 for Lot B. The corresponding LOQ values were consistently higher with 0.096 and 0.085 for Lot A and 0.065 and 0.073 for Lot B. As the LOQ exceeded the cut-off in all assays, the validation parameter for the Detection Limit was successfully passed (see Table 6).

#### 2.2.3. Correctness (Accuracy)

Since the MAT described in Ph. Eur. chapter 2.6.30, Method B, was a semi-quantitative (limit) test method, Accuracy, as defined in USP <1225> and ICH Q2, cannot be demonstrated [15,17]. Instead, we determined Correctness, where one spike concentration with different pyrogens was used instead of three different spike concentrations. The recovery was not analyzed quantitatively as described in the section for Accuracy but relatively based on OD values.

Correctness is defined as the closeness of agreement between the value that is accepted either as a conventional true value or an accepted reference value and the test result (ICH Q2 [17]). For the MAT, we defined Correctness as the closeness of a measured pyrogenicity to the expected pyrogenicity. The latter was assessed by the addition of a defined amount of a pyrogen to an uncontaminated, pyrogen-free sample. Correctness is achieved when the signal of the spiked sample is between the signal obtained from 0.5× and 2× pyrogen spikes (tantamount to the expected pyrogenicity).

**Figure 1:**
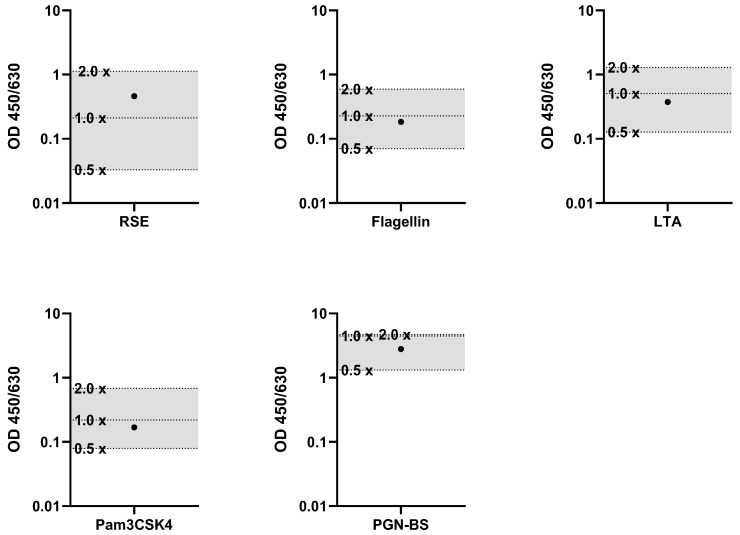
Results of the validation parameter Correctness. Spike recoveries of individual pyrogens in relation to their respective standard dilutions. For each pyrogen (RSE, flagellin, LTA, Pam3CSK4, and PGN-BS), the signal (OD) of the spiked sample (●) was compared to the standard dilution series (0.5×, 1× and 2× standard dilutions). The acceptance range of 50–200% (0.5× and 2×) of the spike concentration is indicated by the shaded area.

To determine the Correctness of the MAT, the signal of a pyrogen-spiked sample was compared to the signal of the corresponding standard dilutions. For this purpose, the cell culture medium, which was used as a sample, was spiked with a defined amount of pyrogen. RSE, flagellin, LTA, Pam3CSK4 and PGN-BS were used as pyrogens. The signals of the sample with the spike and the sample without the spike were examined in relation to the pyrogen standard dilutions. The pyrogen standard dilutions had to frame the concentration which was used for the spike. For the specific acceptance criteria, the signals of the pyrogen standard dilutions had to decrease gradually with increased dilution, and the spike recovery had to be in the range of the OD generated from the 0.5× spike (50%) and 2× spike (200%) of the pyrogen concentration. The results met these acceptance criteria, confirming that the Correctness validation parameter was appropriate. The signals from the spiked samples were within the expected range of 50% and 200% (Figure 1) and the spike recovery for all tested pyrogens fell within the defined range, demonstrating that the MAT’s performance accurately reflected the expected pyrogenicity.

#### 2.2.4. Specificity

Specificity is the ability of the method to unequivocally assess pyrogen contamination in the presence of other components that will be present in the sample. Overall, the MAT is only able to detect pyrogens as a whole. It is not possible to define the exact cause of the pyrogenic contamination. Specificity is given if pyrogens are spiked into a sample and can be determined with acceptable accuracy and precision, showing that the MAT is able to detect various kinds of pyrogens, targeting different TLRs.

The specificity of the MAT was not demonstrated separately; instead, it was evaluated based on the data of Section 2.2.3 (Correctness). The pyrogen spike recovery was between the OD generated from 50% and 200% of the pyrogen concentration, showing a specific response of the MAT toward endotoxins and the tested pyrogens. Therefore, the validation parameter Specificity was passed.

#### 2.2.5. Precision

Precision is defined as the closeness of agreement between the variation in replicates of a series of analysis. For MAT, the Precision was expressed by measuring the intra- and inter-assay coefficient of variation (CV).

(i) Intra-assay Precision (Repeatability)

Intra-assay Precision, also known as repeatability, is defined as the consistency of replicate measurements of a single sample tested under identical conditions within a single assay. To evaluate the intra-assay Precision of the MAT, endotoxin and the non-endotoxin pyrogens defined for the validation (see Section 2.2) were tested as pyrogen samples, and the CVs within a single plate were assessed. For this purpose, three different dilutions of the five pyrogens were tested within one assay. The specific acceptance criterion for intra-assay Precision was that the CV of the pyrogen samples had to be equal or below 30% for each of the tested pyrogens. The results met this acceptance criterion, confirming that the intra-assay Precision validation parameter was passed, as shown in Table 7.

(ii) Inter-assay Precision (ruggedness/intermediate Precision)

Inter-assay Precision, also known as ruggedness/intermediate Precision, refers to the variation observed within a laboratory under changing conditions, such as differences in operators, assay days, or equipment. To determine the inter-assay Precision of the MAT, endotoxin and non-endotoxin pyrogens were tested as pyrogen samples by two different operators, and the CVs of each series of pyrogens were assessed. For this purpose, three different dilutions of each pyrogen sample were tested in one assay.

To facilitate a comparison of results from different assay performances, the obtained OD signals were normalized by transferring them to IL-6 concentration using an IL-6 standard curve. The inter-assay CV was then calculated based on these IL-6 concentrations for each sample.

The specific acceptance criterion for the inter-assay CV of the pyrogen samples had to be equal or below 45%. This criterion was met, confirming that the inter-assay Precision validation parameter was passed, as shown in Table 8.

The acceptance criterion for the inter-assay Precision was defined as CV ≤ 45%, which is higher than the maximum allowed CV of the measurement of one sample (Intra-assay Precision), which was 30% (see above). In the validation parameter inter-assay Precision, not only does the variation in one sample has to be considered, but in addition the assay-to-assay variation has to be considered as well.

#### 2.2.6. Robustness

Robustness is a measurement of the method’s capacity to remain unaffected by small but deliberate variations in the method. In a biological assay like the MAT, there are many parameters that can influence the outcome; therefore, an extensive robustness study was performed to ensure the method runs reliably during routine usage. In total, five different parameters were evaluated.

(i) Lot-to-Lot Comparability of the HaemoMAT^®^ kit

The spike recovery of endotoxin and four NEPs was assessed using two different HaemoMAT^®^ lots. The first criterion was that the signals of the pyrogen standard should decrease gradually with increasing dilution. The second criterion stipulated that the spike recovery has to be between the OD generated from 50% and 200% of both the pyrogen and endotoxin concentration.

**Figure 2:**
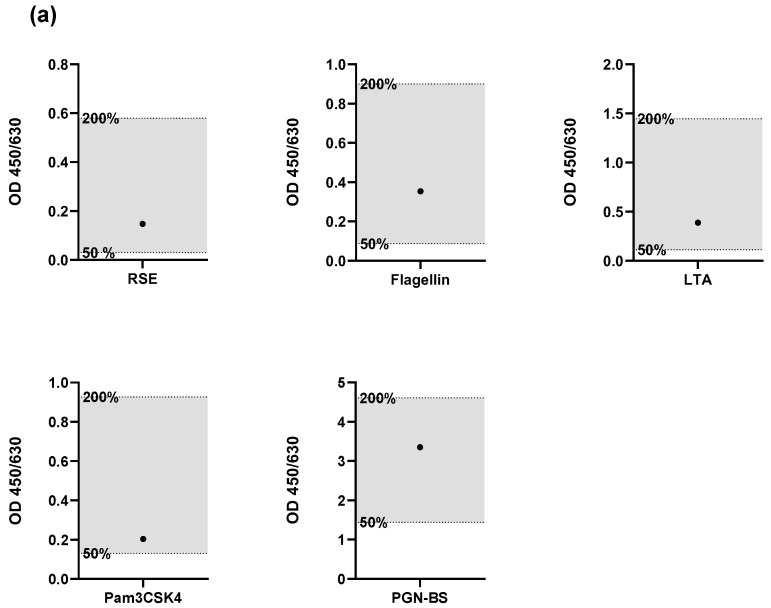

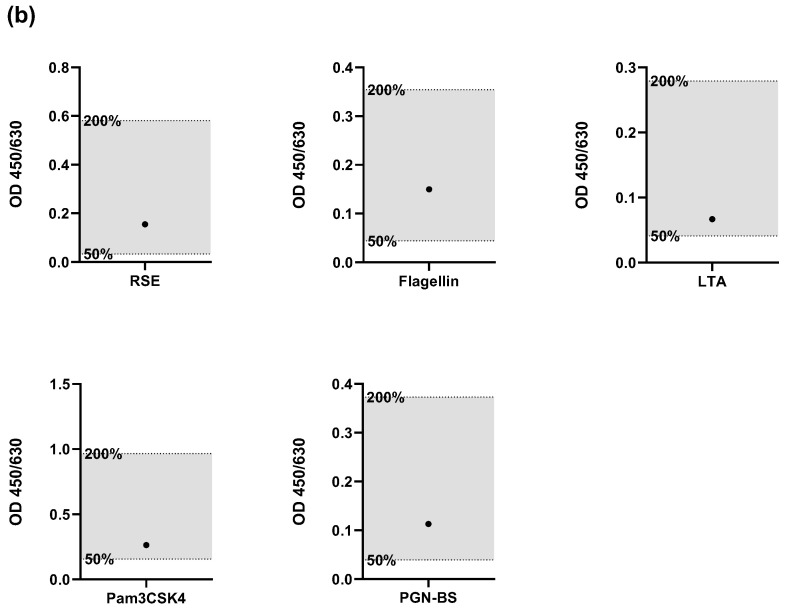
Results for the validation parameter Robustness. Spike recoveries of endotoxin and four non-endotoxin pyrogens (NEPs) assessed using two different HaemoMAT^®^ kit lots: (**a**) Lot A and (**b**) Lot B. For each pyrogen (RSE, flagellin, LTA, Pam3CSK4, and PGN-BS), the OD signal of the spiked sample (●) was compared to the OD range defined by the 0.5× and 2× pyrogen standard concentrations. The acceptance range of 50–200% (0.5× and 2×) of the spike concentration is indicated by the shaded area.

The results, shown in Figure 2, met all of these acceptance criteria, confirming that the lot-to-lot comparability validation parameter was passed. This indicates that the performance of the HaemoMAT^®^ system remained consistent across different lots, ensuring reliable results regardless of the batch used.

(ii) Robustness of the IL-6 measurement over time

**Figure 3:**
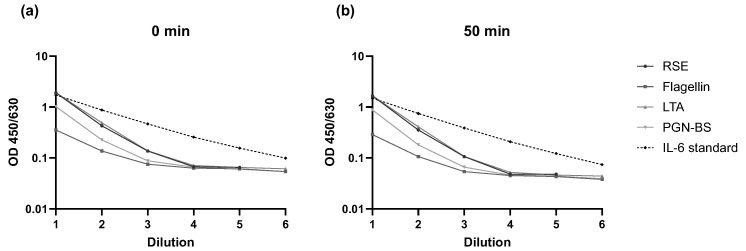
Results of the assessment of Robustness of IL-6 measurement over time. OD signals for each pyrogen (RSE, flagellin, LTA, Pam3CSK4, and PGN-BS) and IL-6 at (**a**) 0 min and (**b**) 50 min after addition of the stop solution in relation to respective dilutions are shown.

According to the manufacturer’s instructions, the IL-6 ELISA should be read within 30 min after addition of the stop solution to the substrate. In this study, endotoxin and three non-endotoxin pyrogens (NEPs) were tested in the MAT. Following the addition of the stop solution, OD measurements for IL-6 were performed every 10 min over the course of one hour to assess signal stability. Additionally, an IL-6 standard curve diluted in assay diluent was included on the ELISA plate to evaluate the direct impact of extended reading time on the IL-6 signal. The specific acceptance criterion was that the signals of the pyrogen samples and the IL-6 standard dilutions should increase gradually with decreasing dilution. This criterion was met. However, the run acceptance criterion (STD4 > STD5) was only fulfilled for the time points between 0 and 50 min after addition of the stop solution. Representative results for the 0 min and 50 min time points are shown in Figure 3. Full data for each time point are provided in the Supplement S1. Ideally, the IL-6 ELISA should be read within 30 min as recommended by the kit manufacturer. However, based on this validation parameter, the assay was robust enough to allow measurement within a time window of 0 to 50 min.

(iii) Robustness of thawing of PBMCs

Typically, the PBMCs are transferred into the cell culture plates within 10 min after thawing. To identify any possible variations in the stimulation pattern or the background signal, the timing from the thawing of the PBMCs until transferring them into the cell culture plate (pre-incubation) was intentionally varied in timesteps of 10 min, 30 min, and 60 min. A dilution series of the RSE was added to the culture plates, which was followed by the cells with indicated pre-incubation times. The purpose of this experiment was to find the maximum allowable pre-incubation time, where all run acceptance criteria (Table 14) are fulfilled. For up to 30 min of pre-incubation time, all run acceptance criteria were fulfilled. With 60 min pre-incubation, the acceptance criteria Cut-Off < LOD did not pass. As a result, it had to be ensured that the pre-incubation time did not exceed 30 min for routine testing.

(iv) Robustness of stimulation time of PBMCs

**Figure 4:**
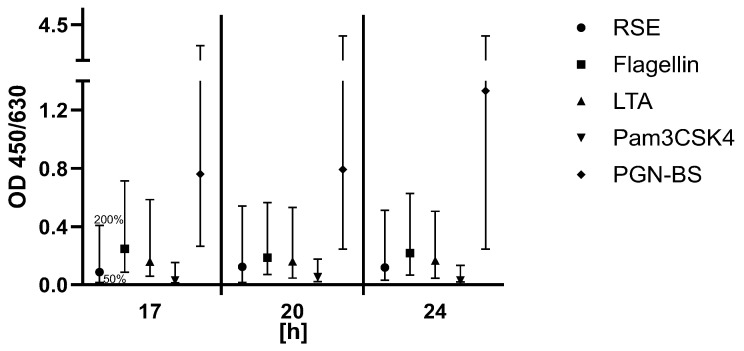
Results for the validation parameter Robustness of stimulation time of PBMCs. For each pyrogen (RSE, flagellin, LTA, Pam3CSK4, and PGN-BS), the OD signal of the spiked sample was compared to the OD range defined by the 0.5× and 2× pyrogen standard concentrations. The acceptance range of 50–200% (0.5× and 2×) of the spike concentration is indicated by the whiskers.

According to the manufacturer’s instructions, the stimulation time of the PBMCs must be 18 ± 1 h at 37 °C. To identify how the stimulation time affects the signal, the stimulation time of PBMCs was intentionally varied in timesteps of 17 h, 20 h, and 24 h. The signals of samples with and without the spikes of five pyrogens in total were examined in relation to the dilution series of the RSE. The PBMCs were thawed, diluted in cell culture medium and pooled. The cells were subsequently transferred into separate cell culture plates and analyzed at their distinct time points. The purpose of this experiment was to find the maximum stimulation time where all acceptance criteria are still met. The specific acceptance criteria were the same as those for (i) lot-to-lot comparability. The signals of the pyrogen standard dilutions had to decrease gradually with increased dilution, and spike recovery had to be between the OD generated from 50% and 200% of the pyrogen and endotoxin concentration (Figure 4).

Criteria were met for all time points between 17 h and 24 h. Ideally, the stimulation time of the PBMCs for routine release testing should have been 18 ± 1 h according to the kit manufacturer, but as a result of this validation parameter, the assay was robust enough to allow stimulation time between 17 h and 24 h.

(v) Robustness of thawing of the cell culture plate

According to manufacturer’s instructions, an incubated cell culture plate can be aliquoted and stored at −80 °C for use on a subsequent occasion. To assess the robustness and widen the applicability of the HaemoMAT^®^ a complete cell culture plate was processed directly after incubation and after one and two freeze/thaw cycles, respectively. The signals of samples with and without the spikes of four pyrogens in total were examined in relation to the dilution series of the RSE.

After incubating the cell culture plate, the plate was aliquoted in three equal cell culture plates. One cell culture plate was processed immediately after aliquoting. The other two cell culture plates were frozen at ≤−65 °C. Both frozen cell culture plates were thawed after approximately 18 h. One was processed, whereas the other was frozen again at ≤−65 °C and processed after approximately 18 h. Thawing was performed by incubating the plates at room temperature for 30 min until every well was visually completely thawed. The specific acceptance criteria were the same as those for (i) lot-to-lot comparability. The signals of the pyrogen standard dilutions had to decrease gradually with increased dilution, and spike recovery had to be between the OD generated from 50% and 200% of the pyrogen and endotoxin concentration.

**Figure 5:**
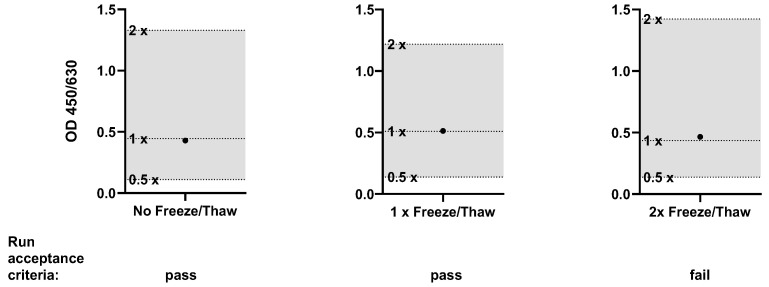
Results for the validation parameter Robustness of thawing of the cell culture plate (LTA only). The OD signal of the spiked sample (●) was compared to the OD range defined by the 0.5× and 2× pyrogen standard concentrations. The acceptance range of 50–200% (0.5× and 2×) of the spike concentration is indicated by the shaded area.

The specific acceptance criteria were met for all plates. Figure 5 shows the resulting OD signal for LTA, and the data from all pyrogens can be seen in the Supplement S2. However, for freezing and thawing of the cell culture plate two times, the run and sample acceptance criteria (Table 14) were not fulfilled (Cut-off). As a result, it must be ensured that the plates can only be processed after a maximum of one freeze/thaw cycle for routine testing.

#### 2.2.7. MAT–RPT Comparison

Particularly as requested by the FDA, a comparative study was conducted to demonstrate the equivalency of the HaemoMAT^®^ system to the RPT. The objective of this study was to assess the suitability of the proposed HaemoMAT^®^ method by comparing the pyrogenicity of various (NEPs) in rabbits, as reported in the published literature, with data generated using the HaemoMAT^®^ kit at concentrations equal to or lower than those used in the RPT. The study assessed responses to different pyrogens, including RSE, LTA, PGN, and Zymosan obtained from the same vendor as described in the literature. It is important to note that all NEP preparations obtained from the different vendors were to some extent contaminated with endotoxins. Hence, additional in-house prepared NEPs were included, which had been shown to be endotoxin-free (Supplement S3).

To allow for a meaningful comparison between both methods, the NEP concentrations used in the MAT were derived from the pyrogenic threshold concentrations that induced fever in rabbits in the RPT studies; see Table 9. For each NEP, four different concentrations, equal to or lower than the concentration determined to be pyrogenic in the RPT, were subsequently tested with the MAT; see Table 9. The concentrations listed for the HaemoMAT^®^ kit represent the final concentration per well, taking into account the dilutions from the addition of the cell culture medium and/or PBMC solution. The comparison was evaluated qualitatively with a positive RPT result corresponding to fever induction in rabbits (pyrogenic threshold dose) as cited in the literature and a positive MAT result defined by an OD above the signal of the Limit of Detection (0.05 EU/mL). The results indicate that NEPs and RSE consistently showed positive results in the MAT at concentrations equivalent to those that produced a positive response in the RPT; see Table 10. Since the NEPs used in the MAT were from the same vendors as those in the RPT studies, it is highly probable that the RPT NEPs were similarly contaminated, suggesting that the true pyrogenic thresholds for NEPs in rabbits might be higher than originally reported.

Specifically for the purchased LTA, endotoxin levels were near the quantification limit at concentration 2. Even at this concentration, which was more than tenfold lower than that tested in the RPT, the MAT yielded a positive signal. While the influence of residual endotoxin cannot be entirely ruled out, the consistent positive outcomes at significantly lower LTA concentrations in the MAT suggest its non-inferiority to the RPT for LTA detection. The results from the in-house, endotoxin-free LTA further underscored the MAT’s sensitivity, demonstrating a clear positive response at concentrations considerably lower than those typically reported for the RPT. This strongly indicates that the MAT’s positive result is attributable to the LTA itself.

For purchased PGN, no endotoxins were detected at concentration 2 (below the quantification limit of the bacterial endotoxins test of 0.05 EU/mL). The MAT generated a positive signal at this concentration, which was more than five times lower than the concentration tested in the RPT, leading to the conclusion that the MAT is superior to the RPT for PGN detection. Similarly, the in-house, endotoxin-free PGN exhibited a positive MAT response at a much lower concentration compared to the RPT, affirming the MAT’s ability to detect PGN directly.

Purchased Zymosan also showed residual endotoxins up to concentration 3. Nevertheless, the MAT yielded a positive signal even at concentration 4, which was more than nine times lower than the RPT-tested concentration. Although residual endotoxin might partially influence the signal, the MAT’s positive outcome at significantly lower concentrations indicates its non-inferiority to the RPT for Zymosan.

Taken together, the MAT–RPT comparison demonstrates that the sensitivity of the HaemoMAT^®^ kit for both endotoxins and NEPs is concluded to be at least non-inferior, if not superior, when compared to the RPT. This finding further supports the reliability of the MAT as a suitable alternative to the RPT, meeting regulatory requirements while offering a modern, animal-free pyrogen testing approach.

### 2.3. Product-Specific Verification

After successful generic validation, which is independent of the product, product-specific feasibility and verification must be tailored to each product individually. Compliance with regulations such as Ph. Eur. 2.6.30 [12] and 2.6.40 [21] is required. According to Ph. Eur. 2.6.30, product suitability testing must assess potential interfering factors, including endotoxins and non-endotoxin pyrogens (NEPs), batch-to-batch comparability, and interference in the detection system.

The product-specific verification was performed using three batches of a pilot product. Data for the product-specific verification of a second product are available in the Supplements S4 and S5. The NEPs LTA and flagellin were included based on potential contamination sources in the manufacturing process, ensuring the coverage of all relevant TLR receptors. The endotoxin limit concentration for the product was defined as 2.5 EU/mL. Using the MAT setup validated during the generic validation, with a sensitivity of 0.05 EU/mL, an MVD of 50 was calculated.

#### 2.3.1. Test for Interfering Factors and Batch-to-Batch Comparability

**Figure 6:**
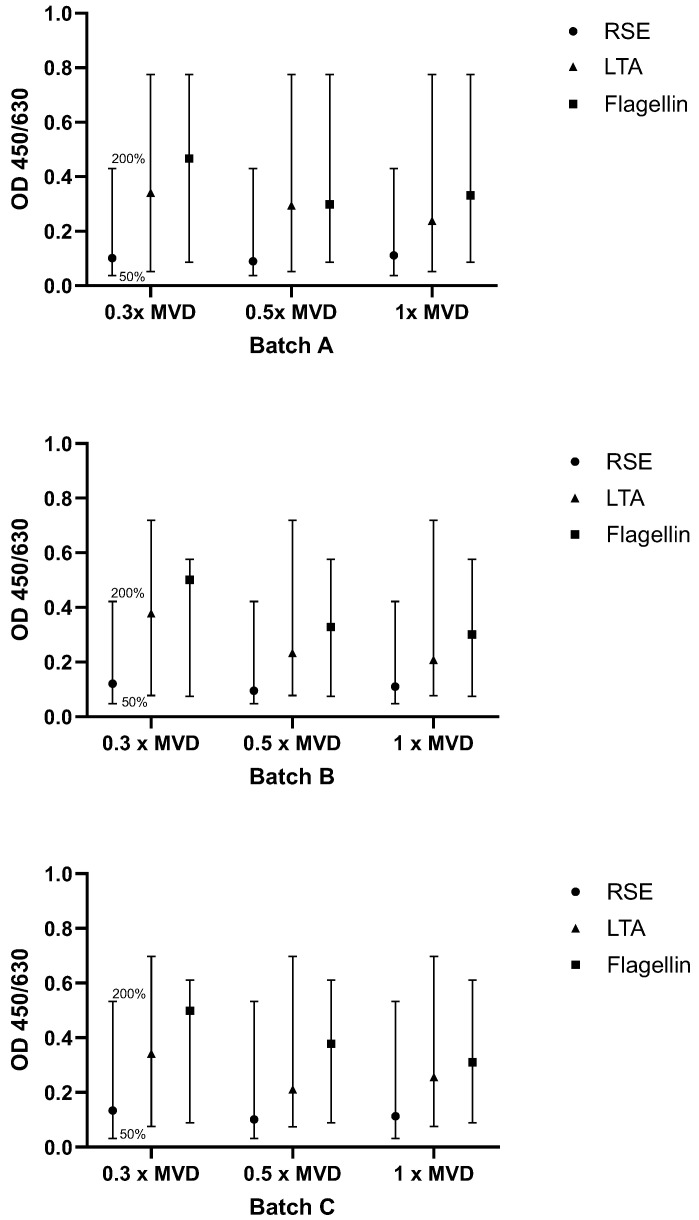
Results for testing interfering factors and batch-to-batch comparability. For each pyrogen (RSE, LTA, flagellin), the OD signal of the spiked sample was compared to the OD range defined by the 0.5× and 2× pyrogen standard concentrations. The acceptance range of 50–200% (0.5× and 2×) of the spike concentration is indicated by the whiskers.

All three product batches were diluted 1:15 (0.3× MVD), 1:25 (0.5× MVD), and 1:50 (1× MVD). Note that the optimal dilutions of the product were determined during feasibility study and might be slightly different for other products. The highest dilution must always equal the MVD. The dilutions were spiked with RSE, flagellin and LTA, and the OD values were compared to samples without spiking (according to Method B). The specific acceptance criteria were that the signals of the pyrogen standard dilutions have to decrease gradually with increased dilution (this only applied to NEP spikes), and spike recovery had to be between the OD generated from 50% and 200% of the endotoxin/NEP concentration. Criteria were met, as shown in Figure 6, indicating that the product did not interfere with pyrogen recovery.

#### 2.3.2. Interference in the Detection System

In the next step, the acceptable lowest dilution of the preparation which was determined in Section 2.3.1 has to be verified to show no interference with the detection system. For this purpose, the 1:15 (0.3× MVD) dilution of the product was diluted 1:4 in cell culture medium, mimicking the assay’s actual testing environment as closely as possible. This pre-dilution was added to a dilution series of IL-6 and analyzed in the IL-6 ELISA. The signals of IL-6 in the sample dilutions were compared to corresponding IL-6 standard dilution. For the specific acceptance criteria, recovery had to be between 80% and 120% of the IL-6 concentration. Table 11 shows the resulting OD values, including recoveries ranging between 94% and 99%. Therefore, the criteria were met, indicating that the product did not interfere with the read-out system.

### 2.4. Release Testing

The release testing of three process performance qualification (PPQ) batches confirmed the absence of detectable pyrogens in the final product (Table 12). The MAT method (including validation) and the corresponding release testing results were submitted to the FDA as part of the regulatory approval process and accepted.

## 3. Discussion

The MAT has emerged as a scientifically robust and ethically preferable alternative to the RPT with the unique advantage of detecting both endotoxins and NEPs. In line with the 3Rs principle and evolving regulatory frameworks, the Ph. Eur. is actively phasing out the RPT. As outlined in the new chapter 5.1.13 on pyrogenicity [14], the MAT is expected to become the default method for pyrogen testing in Europe by 2026. Since it is already compendial under Ph. Eur. chapters 2.6.30 [12] and 2.6.40 [21], implementation requires only a product-specific verification of method suitability. In contrast, the MAT is not yet described as a pharmacopeial procedure in the United States. Hence, per USP <1225> [15], a full method validation as an alternative procedure remains mandatory. This regulatory divergence presents a practical challenge for manufacturers aiming for global product approval.

To address these differences, this study applied a two-tiered validation strategy consisting of a generic, product-independent validation (primary validation) followed by a product-specific verification. The generic validation was conducted under GMP conditions in alignment with ICH Q2 [17], USP <1225> [15], and the former version of Ph. Eur. 2.6.30 [16] (Method B), which was valid at the time of execution. Validation parameters included Range and Linearity, Detection Limit, Accuracy, Specificity, and Precision. Due to the semi-quantitative nature of the MAT, the Quantitation Limit was not applicable. This approach provides a broadly applicable, transferable framework suitable for international regulatory submissions, particularly in contexts where full method validation is required, such as in the U.S.

In addition to regulatory conformity, the study placed special focus on method robustness, acknowledging the biological variability of primary human immune cells used in the MAT. Critical system parameters were examined, including the lot-to-lot comparability of MAT kits, stimulation time, cytokine read-out timing, and freeze–thaw stability of stimulated cell culture plates. The results showed that IL-6 responses remained stable across conditions with consistent detection performance even when read-outs were delayed or stimulation times varied. These findings confirm the assay’s reliability under practical laboratory conditions and support its suitability for routine quality control.

Following generic validation, the method was applied to specific parenteral products in accordance with Ph. Eur. 2.6.30. Interference testing confirmed that neither the formulation matrix nor batch variability interfered with pyrogen detection or cytokine read-out. The resulting data supported a successful Biologics License Application (BLA) submitted to the FDA, where the MAT was accepted as a replacement for the RPT.

At the time of the study, the MAT was validated according to Method B of the former Ph. Eur. chapter 2.6.30, which distinguished three formats: Method A (quantitative), Method B (semi-quantitative, used here), and Method C (reference lot comparison for inherently pro-inflammatory products). In the revised version, these were consolidated into two approaches: Method 1, a unified semi-quantitative format integrating Methods A and B, and Method 2, corresponding to the former Method C for use in inherently pyrogenic products. In addition, the new chapter 2.6.40 specifically addresses MAT testing for vaccines containing inherently pyrogenic components (e.g., outer membrane vesicles, lipidated proteins).

In this context, it is helpful to compare the present study with the work of Daniels et al. [22], who conducted a product-specific GMP verification of MAT Method A for three monoclonal antibodies. Their study focused on quantitative curve evaluation and defined NEP responses under standardized conditions. In contrast, our study provides a comprehensive, generic dataset and includes additional robustness testing relevant for routine use as well as comparison to the traditional RPT showing the equivalence and even superiority of the MAT. While Daniels et al. [22] provide detailed product-level data, our work serves as a methodological reference for broader implementation and regulatory acceptance, especially in jurisdictions like the U.S. that still require full method validation.

The successful implementation of the MAT described here demonstrates that the assay can be applied reliably under GMP conditions and adapted to diverse product types. A generic validation strategy reduces redundancy across products while maintaining the need for product-specific verification to ensure product and matrix compatibility. In Europe, regulatory expectations for MAT implementation are now clearly defined (see Ph. Eur. 2.6.30). In the U.S., however, companies must continue to plan for additional validation efforts, including RPT and BET, at least for PPQ batches of new parenteral products. In the case presented in this paper, the RPT could be avoided, as the MAT was filed and accepted as an alternative method.

Finally, successful MAT adoption requires the careful selection of a suitable test system adapted to product characteristics and regulatory requirements. System performance can vary depending on the assay format, cell source, and read-out parameters. Therefore, robust system qualification, trained personnel, and participation in external proficiency testing are essential to ensure reliability and consistency across laboratories.

In summary, this study provides a validated, transferable strategy for implementing the MAT in pharmaceutical quality control. It supports both European and U.S. regulatory expectations and contributes to the broader goal of replacing animal-based pyrogen testing with a modern, human-relevant assay system. The generated dataset offers practical guidance for laboratories and manufacturers seeking to establish the MAT as a robust and globally accepted alternative to the RPT.

## 4. Materials and Methods

### 4.1. Materials and Reagents

All consumables and reagents were purchased as sterile and pyrogen-free. Unless stated otherwise, all dilutions and samples were prepared in endotoxin-free glass test tubes (in-house). The HaemoMAT^®^ kits (Lot A: 22154, Lot B: 21988) (containing cells, culture media, endotoxin standard and NEP controls) and anti-IL6 ELISA were purchased by Haemochrom Diagnostica GmbH (Essen, Germany). Recombinant Human IL-6 was purchased from BD Bioscience (Franklin Lakes, NJ, USA). RSE was purchased from The European Directorate for the Quality of Medicines & HealthCare (Strassburg, France). Flagellin (*B. subtilis*), lipoteichoic acid (LTA) (*S. aureus*), peptidoglycan (*B. subtilis*) (PGN-BS), and PAM3CSK4 were produced in house (Microcoat Biotechnologie GmbH, Bernried, Germany).

### 4.2. Methods

For each assay, standards, samples, spikes and controls had to be prepared freshly on each working day. They could only be used for more than one assay if the assays were performed in parallel.

#### 4.2.1. Preparation of Endotoxin Standard Dilutions

For endotoxin, standard dilutions of RSE were used. RSE was dissolved in the amount of LRW as printed on the label to a concentration of 2000 EU/mL and vortexed for 30 min at 1400 rpm. Endotoxin standard dilutions were diluted in cell culture medium. RSE dilutions are shown in Table 13. The LOQ for this assay setup was defined with 0.05 EU/mL.

#### 4.2.2. Preparation of NEP Standard Dilutions

For the NEP standard, the kit internal NEPs were used. Each NEP vial was dissolved in cell culture medium as defined within the incoming goods control of the HaemoMAT^®^ kit. Incoming goods control of the HaemoMAT^®^ kit included quality control of the kit and the titration of NEPs for the determination of spike concentration (the signal of NEP at the spike concentration had to be close to the signal of the 2× LOQ endotoxin standard dilution 0.10 EU/mL). The 0.5× spike standard dilution and the 1× spike standard dilution were prepared as 1:4 and 1:2 dilutions of the 2× spike standard dilution. Dilutions were prepared in cell culture medium and were mixed by pipetting up and down 10 times.

#### 4.2.3. Preparation of Endotoxin and NEP for Spike Solutions

For the investigation of interference, endotoxin and NEPs for spiking the samples were prepared. Therefore, endotoxin (RSE) was solubilized in endotoxin-free water LRW (as mention above) and diluted for the endotoxin spike solution in cell culture medium. The internal NEPs were solubilized and diluted in cell culture medium. All dilutions were mixed by pipetting up and down 10 times. For evaluation of the endotoxin spike recovery, the corresponding endotoxin standard dilutions according to Table 13 were used. NEP standard dilutions were prepared from the same solution with concentrations of 2× spike, 1× spike and 0.5× spike. These three solutions were needed for evaluation of the NEP spike recovery in the spiked sample.

#### 4.2.4. Preparation of NEP Samples (In-House NEPs)

In-house NEPs were used as samples. Preparation was the same as the preparation of NEPs for standard dilutions, but obtained values were evaluated as samples instead of controls. NEP vials were dissolved in cell culture medium and mixed by pipetting up and down 10 times.

#### 4.2.5. Monocyte Activation Test

The general performance of the MAT assay is based on the manufacturer’s instructions. Only the sample-to-PBMC ratio and corresponding concentrations were adapted for the validation according to method development.

For MAT, 50 µL of the samples, PPCs and the prepared standard curve dilutions were added in quadruplets to a 96-well microtiter plate. For the spiking of samples, prepared endotoxin and pyrogen spike solutions (1× spike) were added into the corresponding wells of the cell culture plate (50 µL). For unspiked samples, 50 µL of the cell culture medium was added instead of the spiking solution. Thawed PBMCs were then diluted in pre-warmed cell culture medium, and 100 µL of cell suspension (PBMC) was added per well of the 96-well plate. The plate was incubated at 37 °C, 5% CO_2_ for 17 h to 19 h. After the stimulation of PBMCs, the whole cell culture plate was stored at ≤−65 °C or directly used for the performance of an anti-IL-6 ELISA (part of the HaemoMAT^®^ kit).

A defined volume of dilution buffer, as specified for the respective kit lots in the certificate of analysis of the HaemoMAT^®^ kit, was added to each well of the anti-IL-6 ELISA plate. Where applicable, the dilution buffer was replaced by 200 µL of the IL-6 control provided with the kit. Subsequently, the lot-specific volume of PBMC suspension (as indicated in the CoA of the corresponding MAT lot) was added to each well. The plate was incubated for one hour, at room temperature on an orbital shaker (~700 rpm), allowing the IL-6 released by pyrogen-stimulated PBMCs to bind to the immobilized capture antibody. After incubation, the plate was washed manually five times with the buffer supplied with the kit to remove unbound components. An enzyme-linked monoclonal anti-IL-6-detection antibody was then added, which was followed by incubation for one hour at room temperature on an orbital shaker (~700 rpm). A second manual washing step (5×) was performed to remove unbound conjugate. TMB substrate solution (provided as part of the kit) was added to each well, and the plate was incubated for 30 min in the dark at room temperature on an orbital shaker (~700 rpm). Color development was proportional to the amount of IL-6 bound to the capture antibody. The enzymatic reaction was stopped by addition of the stop solution after exactly 30 min, and the intensity of the color was measured at 450–630 nm with a BioTek ELX808 absorbance microplate reader (BioTek Instruments, Inc., Winooski, VT, USA) coupled with Gen5 Secure software (version 3.11, BioTek), which was used for the calculation of statistical characteristics. For the summarization and visualization of data, GraphPad Prism 10.1.1 was used.

#### 4.2.6. Statistical Analysis

We determined the MVD using the following expression:

$$MVD=\frac{CLC\;\times \;C}{LOD}$$

CLC = contaminant limit concentration: 2.5 EU/mL;
C = concentration of test solution: 1;
LOD = limit of detection.

Please note that in the updated section (Supplement 11.5 [12]), the LOD was replaced by test sensitivity, which is the lowest endotoxin reference standard concentration on the standard curve whose response exceeds the cut-off value.

The following descriptive statistics were calculated:

(1) $$Mean:\bar{x}=\;\frac{\sum_{i=1}^{n}x_{i}}{n}\;$$

All run acceptance criteria and specific criteria were calculated based on the mean of the OD (450–630 nm).

(2) $$Standard\;deviation\;\sigma =\sqrt{\frac{\;\sum {\left(x-\bar{x}\right)}^{2}}{n-1}}$$

(3) $$Coefficient\;of\;variation\;\left(\%\right):CV=\left(\frac{\sigma }{\bar{x}}\right)\times 100$$

Newton−Raphson 4−parameter logistic function:

(4) $$y=\left(\frac{A-D}{1+({x/c)}^{B}}\right)+D$$

(5) $$Cut\text{-}off\;value=\bar{x}\left(Blank\right)+3\;\sigma \;(Blank)$$

n = number of values, x = value of sample,$\;\bar{x}$ = mean value, i = index for individual values, σ = standard deviation; A, B, C and D = parameters for curve fitting.

Samples and PPCs, which were above the signal of the LOD, had to have CVs equal or below 30%. CVs were calculated on the basis of at least three OD values, after elimination of the outlier (Dixon α = 0.1). If the mean OD of the sample or PPC (sample + spike) was below the OD of the LOD, a CV calculation was not performed and no acceptance criterion regarding the CV had to be passed. The acceptance criterion regarding the CV is not part of the requirements of Ph. Eur. 2.6.30. This criterion was set up to exclude values with high variation.

For all assays in this method validation run, the acceptance criteria and sample acceptance criteria were defined to ensure the assay’s validity (see Table 14 and Table 15). Additionally, specific criteria for describing the validity of the individual validation and verification parameters were defined.

For all parameters, the acceptance criteria were met (unless stated differently).

## Figures and Tables

**Table 1:** Spike controls as reference for valid PPC recoveries of individual pyrogens in example product, expressed in EE/mL values. These were set to 100% for 50–200% calculation of the PPC recoveries.

[EE/mL]	1 × Spike	
RSE	0.099	100%
LTA	0.093	100%
Flagellin	0.133	100%

**Table 2:** PPC recoveries, expressed as percent values.

Dilution	1:37.5 (0.25 × MVD)	1:75 (0.5 × MVD)	1:150 (1 × MVD)
RSE	73%	80%	81%
LTA	128%	117%	119%
Flagellin	115%	116%	117%
**Status**	**Valid**	**Valid**	**Valid**

**Table 3:** Spike controls as reference for valid PPC recoveries of individual pyrogens in example product, expressed in OD (450–630 nm) values. The OD value of the blank is subtracted from the OD value of the pyrogens.

OD (450–630 nm)	0.5 × Spike (=50% Recovery)	2 × Spike (=200% Recovery)
RSE	0.029	3.305
LTA	0.051	0.707
Flagellin	0.15	1.26

**Table 4:** PPC recoveries, expressed as OD (450–630 nm) values. The OD value of the unspiked sample is subtracted from the OD value of the pyrogen spike for three different dilutions of the individual pyrogens.

Dilution	1:37.5 (0.25 × MVD)	1:75 (0.5 × MVD)	1:150 (1 × MVD)
Sample (without spike)	0.02	0.015	0.015
RSE	0.08	0.11	0.11
LTA	0.33	0.26	0.28
Flagellin	0.64	0.66	0.67
**Status**	**Valid**	**Valid**	**Valid**

**Table 5:** OD values for the RSE standard curve used for evaluation of the validation parameters Range and Linearity.

RSE Standard Concentration	OD
0.40 EU/mL	2.663
0.20 EU/mL	0.629
0.10 EU/mL	0.128
0.050 EU/mL	0.065
0.025 EU/mL	0.062

**Table 6:** Specific acceptance criteria and results for the validation parameter Detection Limit using different lots of the HaemoMAT^®^ kit.

Lot Assay	A 1	A 2	B 1	B 2
LOQ (OD (RSE 0.050 EU/mL))	0.096	0.085	0.065	0.073
Cut-off (OD (Blank) + 3 × SD OD (Blank))	0.080	0.064	0.037	0.052
Specific acceptance criterion (Pass/ Fail)	Pass	Pass	Pass	Pass

**Table 7:** Specific acceptance criteria and results for the validation parameter intra-assay Precision.

Pyrogen		Intra-Assay CV [%]	
	0.5× Dilution	1× Dilution	2× Dilution
RSE	6	7	12
Flagellin	6	3	6
LTA	8	8	11
Pam3CSK4	9	7	10
PGN-BS	15	6	10

**Table 8:** Specific acceptance criteria and results for the validation parameter inter-assay Precision.

Pyrogen		Inter-Assay CV [%]	
	0.5× Dilution	1× Dilution	2× Dilution
RSE	32	29	31
Flagellin	27	18	30
LTA	26	29	40
PGN-BS	13	9	32

**Table 9:** Literature-based comparison of pyrogen doses in RPT and selected MAT concentrations for MAT–RPT comparison.

Pyrogen	Exposure Dose Leading to Fever in Rabbits	Corresponding Concentration in the MAT ^a^	NEP Concentrations Tested Using the HaemoMAT^®^			
			Conc. 1	Conc. 2	Conc. 3	Conc. 4
Reference Standard Endotoxin [18]	Administered: 10 EU/kg rabbit bodyweight Normalized: 0.18 mg/mL rabbit blood ^a^	≤0.18 EU/mL (corresponds to ≤0.0000216 mg/mL)	0.1 EU/mL ^b^	0.05 EU/mL ^c^	0.025 EU/mL ^c^	0.0125 EU/mL ^c^
LTA [18,19]	Administered: 20 mg/kg rabbit bodyweight Normalized: 0.35 mg/mL rabbit blood ^a^	≤0.35 mg/mL	0.350 mg/mL ^b^	0.030 mg/mL ^d^	0.015 mg/mL ^d^	0.0075 mg/mL ^d^
	Administered: 75 mg/kg rabbit bodyweight Normalized: 1.315 mg/mL rabbit blood ^a^					
PGN [18]	Administered: 35 mg/kg rabbit bodyweight Normalized: 0.61 mg/mL rabbit blood ^a^	≤0.61 mg/mL	0.610 mg/mL ^b^	0.122 mg/mL ^d^	0.061 mg/mL ^d^	0.0305 mg/mL ^d^
Zymosan [16]	Administered: 14 mg/kg rabbit bodyweight Normalized: 0.25 mg/mL rabbit blood ^a^	≤0.25 mg/mL	0.250 mg/mL ^b^	0.125 mg/mL ^d^	0.0625 mg/mL ^d^	0.0313 mg/mL ^d^

^a^ Administered dose was normalized: dividing by the volume of rabbit blood (57 mL/kg rabbit bodyweight) [20]. ^b^ Concentration corresponding to the exposure dose leading to a fever reaction in the RPT or even lower in the case of RSE. ^c^ Concentration corresponding to the RSE standard curve. The RSE standard curve includes an additional concentration 5 at 0.00625 EU/mL. ^d^ Pre-determined concentrations to obtain a better understanding of the sensitivity of the MAT regarding the different NEPs.

**Table 10:** OD signals (450–630 nm) (average of the replicates) of pyrogens tested using HaemoMAT^®^.

Pyrogen	Conc. 1	Conc. 2	Conc. 3	Conc. 4
RSE	3.250	0.560	0.132	0.067
LTA	4.603	0.110	0.052	0.041
LTA (in-house)	4.536	0.190	0.093	0.053
PGN	4.427	0.767	0.301	0.138
PGN (in-house)	4.066	0.322	0.131	0.060
Zymosan	4.464	2.926	1.054	0.322

**Table 11:** Results (OD (450–630) values) for the validation parameter Interference in the detection system.

IL-6 [pg/mL]	OD (IL-6 Standard Concentration)	OD (IL-6 in Pre-Diluted Test Item)	Recovery in Pre-Diluted Test Item [%]
13	0.492	0.479	97
6.3	0.273	0.257	94
3.1	0.169	0.167	99
0	0.072	0.069	-

**Table 12:** Summary of results from release testing using the MAT.

Batch	Results	RSE PPC
1	OD < LOQ	Pass
2	OD < LOQ	Pass
3	OD < LOQ	Pass

**Table 13:** RSE standard dilutions and PPC concentration.

Endotoxin Standard Dilutions [EU/mL]	PPC Concentration [EU/mL]
0.40 (=8× LOQ), 0.20 (=4× LOQ), 0.10 (=2× LOQ), 0.05 (=1× LOQ), 0.025 (=0.5× LOQ)	0.10

**Table 14:** Run acceptance criteria.

Type	Run Acceptance Criteria
Endotoxin standard dilution	The signals of the endotoxin standard dilutions must decrease gradually with increased dilution ^1^
Blank	The signal of the blank must be equal to or below OD 0.10 ^1^
Cut-off value of the blank	The cut-off value of the blank (corresponding to LOD) must be below the signal of the LOQ (=OD (0.05 EU/mL)) ^1^
NEP controls	The signals of the NEP controls must be above the signal of the 2× LOQ endotoxin standard dilution (0.10 EU/mL) ^2^
IL-6 control	The signal of the IL-6 control must be above OD 1.8 ^2^
Signal to noise	The signal-to-noise ratio of the 2× LOQ endotoxin standard dilution (0.10 EU/mL) must be below 100 ^2^
CV	The CV of standards and controls must be ≤30%

^1^ Requirement according to Ph. Eur. 2.6.30. ^2^ Based on requirement according to HaemoMAT^®^ manual.

**Table 15:** Sample acceptance criteria.

Type	Sample Acceptance Criteria
CV	The CV of samples and PPC must be ≤30%
Spike recovery	OD of samples spiked with endotoxins or non-endotoxin pyrogens must be 50% to 200% of the respective standard dilution.

## Data Availability

The original contributions presented in this study are included in the article/Supplementary Materials. Further inquiries can be directed to the corresponding author.

## Supplementary Materials

The following supporting information can be downloaded at https://www.mdpi.com/article/10.3390/ijms262211136/s1.
