# Supplementary material for: Validation of the Monocyte Activation Test Demonstrating Equivalence to the Rabbit Pyrogen Test
Source: Int J Mol Sci. 2025 Nov 18;26(22):11136. doi: 10.3390/ijms262211136 (PMC12652761; doi:10.3390/ijms262211136)
Supplement: Supplementary file 1 [file ijms-26-11136-s001.zip › ijms-3932338-supplementary.pdf]

Supplement S1. Results for the validation parameter Robustness of IL-6 measurement over time. OD signals for each pyrogen (RSE, flagellin, LTA, Pam3CSK4, and PGN-BS) and IL-6 at 0 min - 60 min after addition of the stop solution in relation to respective dilutions are shown.

Pyrogen	Dil. 1	Dil. 2	Dil. 3	Dil. 4	Dil. 5	Dil. 6 (only NEP and IL-6)	Acceptance criterion: decreasing signal with increasing dilution
0 min							
RSE	1.909	0.429	0.137	0.067	0.065	NA	Pass
Flagellin	0.353	0.137	0.075	0.063	0.061	0.054	Pass
LTA	1.906	0.492	0.138	0.071	0.065	0.061	Pass
PGN-BS	1.023	0.226	0.088	0.066	0.060	0.054	Pass
IL-6 standard	1.718	0.875	0.467	0.257	0.156	0.099	Pass
10 min							
RSE	1.867	0.416	0.131	0.063	0.062	NA	Pass
Flagellin	0.342	0.131	0.070	0.060	0.057	0.051	Pass
LTA	1.866	0.479	0.133	0.067	0.061	0.058	Pass
PGN-BS	0.999	0.218	0.084	0.062	0.056	0.051	Pass
IL-6 standard	1.678	0.854	0.453	0.249	0.150	0.095	Pass
RSE	1.817	0.402	0.125	0.059	0.058	NA	Pass
Flagellin	0.329	0.125	0.066	0.057	0.054	0.048	Pass
LTA	1.818	0.463	0.128	0.063	0.057	0.055	Pass
PGN-BS	0.971	0.210	0.081	0.059	0.054	0.048	Pass
IL-6 standard	1.631	0.829	0.437	0.239	0.144	0.090	Pass
30 min							
RSE	1.765	0.386	0.119	0.056	0.055	NA	Pass
Flagellin	0.317	0.119	0.063	0.053	0.051	0.044	Pass
LTA	1.763	0.444	0.121	0.060	0.053	0.051	Pass
PGN-BS	0.939	0.200	0.076	0.054	0.051	0.045	Pass
IL-6 standard	1.585	0.802	0.421	0.229	0.137	0.085	Pass
40 min							
RSE	1.714	0.369	0.112	0.052	0.052	NA	Pass
Flagellin	0.303	0.112	0.058	0.048	0.047	0.041	Pass
LTA	1.711	0.426	0.115	0.056	0.049	0.047	Pass
PGN-BS	0.907	0.190	0.071	0.050	0.048	0.042	Pass
IL-6 standard	1.540	0.776	0.404	0.218	0.129	0.080	Pass
50 min							
RSE	1.667	0.353	0.106	0.048	0.048	NA	Pass
Flagellin	0.288	0.106	0.054	0.045	0.043	0.038	Pass
LTA	1.663	0.408	0.107	0.052	0.046	0.044	Pass
PGN-BS	0.876	0.180	0.066	0.046	0.044	0.039	Pass
IL-6 standard	1.496	0.749	0.387	0.208	0.122	0.074	Pass
60 min - Run Acceptance Criteria did not pass (STD4 not > STD5)							
RSE	1.623	0.338	0.099	0.044	0.044	NA	Assay failed
Flagellin	0.275	0.099	0.050	0.042	0.039	0.035	Assay failed
LTA	1.618	0.391	0.101	0.049	0.042	0.040	Assay failed
PGN-BS	0.847	0.170	0.062	0.043	0.040	0.036	Assay failed
IL-6 standard	1.456	0.725	0.371	0.197	0.115	0.070	Assay failed

Supplement S2. Results for the validation parameter Robustness of Thawing of the Cell Culture Plate (The OD signal of the spiked sample was compared to the OD range defined by the 0.5× and 2× pyrogen standard concentrations. The acceptance range of 50–200% (0.5x and 2x) of the spike concentration is indicated by the shaded area.

Pyrogen	OD 1 x Pyrogen Sample	OD 0.5 x Pyrogen Standard	OD 1 x Pyrogen Standard	OD 2 x Pyrogen Standard	Acceptance criterion: spike recovery
No Freeze/Thaw cycle					
RSE	0.459	0.041	0.188	1.024	Pass
Flagellin	0.188	0.048	0.193	0.715	Pass
LTA	0.429	0.111	0.446	1.329	Pass
Pam3CSK4	0.167	0.066	0.217	0.744	Pass
PGN-BS	2.783	1.199	4.139	4.633	Pass
1 Freeze/Thaw cycle					
RSE	0.549	0.049	0.234	1.216	Pass
Flagellin	0.212	0.063	0.225	0.681	Pass
LTA	0.513	0.138	0.509	1.218	Pass
Pam3CSK4	0.199	0.092	0.279	0.746	Pass
PGN-BS	3.380	1.478	4.268	4.721	Pass
2 Freeze/Thaw cycle - Run Acceptance Criteria did not pass (Cut-off)					
RSE	0.516	-0.004	0.196	0.971	Assay failed
Flagellin	0.217	0.062	0.221	0.659	Assay failed
LTA	0.466	0.138	0.435	1.423	Assay failed
Pam3CSK4	0.163	0.089	0.260	0.814	Assay failed
PGN-BS	3.208	1.473	4.225	4.621	Assay failed

Supplement S3. Endotoxin content determined in NEP solutions

NEP	Conc. 1	Conc. 2	Conc. 3	Mean endotoxin contamination in the NEP preparation
LTA	0.67 EU/mL	0.053 EU/mL	-	0.46 EU/μg
PGN	0.202 EU/mL	< 0.05 EU/mL	-	0.08 EU/μg
Zymosan	4.09 EU/mL	1.76 EU/mL	0.761 EU/mL	1.64 EU/μg
LTA (in-house)	< 0.05 EU/mL	< 0.05 EU/mL	-	< 0.05 EU/mL
PGN (in-house)	< 0.05 EU/mL	-	-	< 0.05 EU/mL

Supplement S4. Product specific verification for a second product. Results for the validation parameter Test for interfering factors (endotoxin and NEPs) and batch-to-batch comparability. For each pyrogen (RSE, LTA, Flagellin), the OD signal of the spiked sample was compared to the OD range defined by the 0.5× and 2× pyrogen standard concentrations.

	Pyrogen	OD 1 x Pyrogen Sample (product)	OD 0.5 x Pyrogen Standard	OD 1 x Pyrogen Standard	OD 2 x Pyrogen Standard	Acceptance criterion: spike recovery
Batch A	0.25 x MVD					
	RSE	0,199	0,055	0,195	1,078	Passed
	Flagellin	0,826	0,131	0,354	1,096	Passed
	LTA	0,473	0,105	0,297	0,985	Passed
	0.5 x MVD					
	RSE	0,230	0,055	0,195	1,078	Passed
	Flagellin	0,528	0,131	0,354	1,096	Passed
	LTA	0,407	0,105	0,297	0,985	Passed
	1 x MVD					
	RSE	0,224	0,055	0,195	1,078	Passed
	Flagellin	0,537	0,131	0,354	1,096	Passed
	LTA	0,352	0,105	0,297	0,985	Passed

Batch B	0.25 x MVD					
	RSE	0,149	0,061	0,155	1,105	Passed
	Flagellin	1,524	0,273	0,719	1,653	Passed
	LTA	0,509	0,090	0,358	1,091	Passed
	0.5 x MVD					
	RSE	0,151	0,061	0,155	1,105	Passed
	Flagellin	0,986	0,273	0,719	1,653	Passed
	LTA	0,424	0,090	0,358	1,091	Passed
	1 x MVD					
	RSE	0,183	0,061	0,155	1,105	Passed
	Flagellin	1,038	0,273	0,719	1,653	Passed
	LTA	0,314	0,090	0,358	1,091	Passed

Batch C	0.25 x MVD					
	RSE	0,178	0,048	0,164	0,998	Passed
	Flagellin	0,764	0,152	0,393	1,138	Passed
	LTA	0,417	0,082	0,236	0,735	Passed
	0.5 x MVD					
	RSE	0,165	0,048	0,164	0,998	Passed
	Flagellin	0,645	0,152	0,393	1,138	Passed
	LTA	0,307	0,082	0,236	0,735	Passed
	1 x MVD					
	RSE	0,200	0,048	0,164	0,998	Passed
	Flagellin	0,564	0,152	0,393	1,138	Passed
	LTA	0,265	0,082	0,236	0,735	Passed

Supplement S5. **Product specific verification for a second product.** Results (OD values) for the validation parameter Interference in the detection system

IL-6 [pg/mL]	OD (IL-6 standard concentration)	OD (IL-6 in pre-diluted test item)	Recovery in pre-diluted test item [%]
13	0.458	0.465	102
6.3	0.236	0.235	100
3.1	0.129	0.125	97
0	0.015	0.014	-
